# Polio eradication in Nigeria: evaluation of the quality of acute flaccid paralysis surveillance documentation in Bauchi state, 2016

**DOI:** 10.1186/s12889-018-6185-z

**Published:** 2018-12-13

**Authors:** Adamu Ibrahim Ningi, Faisal Shuaib, Luka Mangveep Ibrahim, Jalal-Eddeen Abubakar Saleh, Khalid Abdelrahim, Isah Mohammed Bello, Bashir Abba, Ticha Jonhson Muluh, Fiona Braka, Sisay G. Tegegne, Abdullahi Wallah, Charles Korir, Samuel Bawa, Mahmood Saidu, Peter Nsubuga

**Affiliations:** 1 World Health Organization, Country Representative Office, Abuja, Nigeria; 2 National Primary Healthcare Development Agency, Abuja, Nigeria; 3 Global Public Health Solutions, Atlanta, GA USA

**Keywords:** Acute flaccid paralysis, Surveillance, Certification, Poliomyelitis, Bauchi state

## Abstract

**Background:**

Nigeria is the only country in Africa that is yet to be certified as polio free. Surveillance for acute flaccid paralysis (AFP) is the foundation of the polio eradication initiative since it provides information to alert both health managers and clinician that timely actions should be initiated to interrupt transmission of the polio virus. The strategy also provides evidence for the absence of wild poliovirus. This evaluation was performed to assess key quality indicators defined by the polio eradication program and thus to identify gaps to allow planning for corrective measures to achieve a polio-free situation in Bauchi state and in Nigeria at large. We conducted a cross-sectional descriptive study which involved a desk review of documents to authenticate the correctness and completeness of data, and a review of documented evidence for the quality of AFP surveillance. We interviewed Local Government Authority (LGA) surveillance officers and clinicians from focal and non-focal sites, along with caregivers of children with AFP and community leaders. The data were entered and analyzed in a Microsoft Excel spreadsheet.

**Methods:**

We conducted a cross-sectional study of the AFP surveillance and documentation in eighteen of the twenty Local Government Areas (LGAs) of Bauchi State. We assessed the knowledge of the clinician at focal and non-focal sites on case definition of AFP, the number and method of stool specimen collection to investigate a case and types of training received for AFP surveillance. We verified AFP case investigations for the last three years: The caregivers (mothers) were interviewed to authenticate the reported information of AFP cases, the method used for stool specimen collection and feedbacks. Community leaders’ knowledge on AFP surveillance was also assessed. Data was entered and analyzed in excel spread sheet.

**Results:**

Of the 18 LGA Disease Surveillance and Notification Officers (DSNOs), only 2 (11%) and 5 (28%) had reports of polio outbreak investigations and supervisory visits at the lower levels, respectively. Furthermore, only 6 (33%) and 7 (39%) of the DSNOs had minutes of meetings and surveillance work plans, respectively. Of the 31 AFP cases investigated, only 39, 26, 23, and 23% had correct and complete information for the birth day, birth month, date of onset of paralysis, and date of investigation, respectively. Seventy-one percent of the clinicians at the AFP focal sites knew the correct definition for AFP compared with only 30% at the non-focal sites. Of the 38 caregivers (mothers), 16 (42%) did not remember the day or month the AFP investigation was conducted. However, 95% gave a correct number of stool samples collected and 40% mentioned that the samples were collected 24 h apart. Feedback was not given to 26 (68%) of the caregivers. The majority (79%) of the community leaders knew how to recognize a case of AFP and knew that the stool was the specimen required for the investigation, but 21% did not know to whom they should report a case of AFP in their community.

**Conclusion:**

This study revealed a gap in the quality indicators for polio eradication in the state, especially regarding knowledge and documentation for AFP surveillance at the operational level. Regular training of the DSNOs and focal persons, regular sensitization of clinicians, community education, supplies of reporting tools, and ensuring their judicious use will improve AFP surveillance in the state.

## Background

Poliomyelitis, a disease targeted for eradication in the world at a World Health Assembly (WHA) about three decades ago, still poses a public health challenge [1]. Significant progress has been made in the global quest for the eradication of the disease leading to the certification of four out of the six World Health Organization (WHO) regions as being polio-free; only the WHO Eastern Mediterranean and the African Regions are yet to be certified polio-free. The certification of the WHO Africa region is dependent on Nigeria, while certification of the WHO Eastern Mediterranean region is dependent on Pakistan and Afghanistan [2].

Surveillance for acute flaccid paralysis (AFP) is one of the four strategies for eradication of the polio virus in the world. The strategy provides the information needed to alert health managers and clinicians to initiate timely actions to interrupt transmission of the polio disease [3–6]. The strategy is based on the premise that any AFP case is a potential case of poliomyelitis and should be accorded prompt attention. AFP surveillance in Nigeria is both passive and active [7]. Surveillance data are generated from reporting sites at primary healthcare facilities and hospitals. Each health facility maintains facility registers that document all cases and diagnoses made by the physician or clinician. The data from the reporting sites are collated at the Local Government Area (LGA) by the LGA Disease Surveillance and Notification Officer (DSNO) for onward transmission to the state. The LGA DSNOs have the requisite responsibilities to maintain documented reports and all surveillance activities conducted in the LGA, including the provision of feedback to the lower levels. Community volunteers are also trained, and serve as informants including participating in active searches for cases through home visits or social activities in the communities to complement the facility-based surveillance system for the eradication of polio [8]. The multiple participants make the surveillance system complex. Any incorrect or wrong data, especially at the operational unit, will translate to misinformation and will mislead decision on the entire process for polio eradication in the state and the country, making the quality of data a key concern. The quality of the data and the system operation were identified as critical guiding principles for surveillance of vaccine-preventable diseases by the global framework for immunization monitoring and surveillance [9]. These quality concerns have a bearing on the knowledge about AFP surveillance, including skills of documentation of the officers at all levels [10, 11]. Good and complete documentation is the proxy indication of the quality of the system, while poor documentation translates to the possibility of missing wild poliovirus (WPV) in the past. To achieve the desired results, the quality of the AFP surveillance must not be compromised, especially at the operational units (the health facilities and LGAs). The quality of the data at these levels is critical and determines quality and reliability of the entire surveillance system [12]. We conducted this study to evaluate the quality of the AFP surveillance system to identify gaps and plan for a corrective measure to achieve a polio-free Nigeria.

## Methods

Bauchi State is located in the northeastern part of Nigeria and has an estimated population of 4,653,066, with a total landmass of 49,119 km^2^ [13]. It shares national borders with Gombe, Taraba, and Yobe to the East, Jigawa and Kano to the North, Kaduna to the West, and Plateau to the South. The state has cultural and religious similarities with these neighboring states, thus making it a transit point especially for trans-border activities [14]. Bauchi state is one of the high-risk states for wild poliovirus transmission in the northeast geopolitical zone of Nigeria.

We conducted a cross-sectional study in 150 health facilities in the state (65% designated focal sites for AFP surveillance and 35% non-focal sites) from the 20 LGAs in the state. The sample size for the study was determined by the number of health facilities in the state and those classified as focal sites for AFP surveillance using the formula for sample size calculation (*n* = z_α_^2^pq/d^2^). Where *n* = sample size, z_α_ = 1.96 (95% confidence interval), d = degree of precision of 0.05, p is the proportion of focal sites, and q = 1 – p [15].

The proportion of health facilities designated as focal sites accounted for 9.7% of the health facilities in the state and gave us a minimum sample size of 135 (adding 10% for nonresponses gave a sample size of 150). The respondents for the study were the DSNO, healthcare providers (clinicians) at focal and non-focal sites, caregivers (mothers) of children identified with AFP, and the community leaders.

All the AFP cases identified and investigated between January 2012 and December 2015 were verified using a checklist to validate the completeness, accuracy, and reliability of the data documented during the investigation. The structure available for AFP surveillance and documentation were assessed at the LGA (office of DSNO) and the health facilities, including the availability materials and human resources for AFP surveillance, documented evidence for AFP surveillance, and active case searches. We tested the knowledge of the clinician at focal and non-focal sites on case definition of AFP, stool specimen collection for AFP investigation, and transportation of the specimen. Clinicians in the focal and non-focal sites were assessed on when and where to report a case identified with AFP, including documentation of the AFP case, the type of training they received on AFP surveillance, and the last time they were trained. The non-AFP focal sites (health facilities) were selected by simple random sampling for each LGA. For every health facility selected, only one clinician was interviewed.

The caregivers were interviewed to validate the reported information of AFP cases, their participation in the collection of stool sample specimens, method of collection of the stool samples, feedback on the AFP case that was investigated, and awareness of the existence of other AFP cases in the community using interviewer-administered questionnaires. The community leaders were also interviewed; questions were asked on how to identify a case of AFP and types of specimens required for investigation of AFP cases. Correct responses included mention of fever with sudden onset of paralysis of the limbs in any person aged 15 years or below.

The quantitative data were entered, cleaned, and analyzed in a Microsoft Excel spreadsheet and presented as proportions. The knowledge of the respondents on AFP case definition and stool sample collection were scored based on the number of correct points mentioned by the respondents. A four-point domain for AFP case definition and sample collection and storage were used for scoring, with each element mentioned by the respondents representing one point. Three and four correct points were taken as good and very good knowledge, respectively, while two and below two points were taken as fair and poor knowledge, respectively.

## Results

Review of the surveillance indicators for polio eradication in the state from 2012 to 2016 showed that the last WPV reported in the state was in September 2013. The average timeliness of reporting was 96%, completeness of reporting was 98%, and stool adequacy was 99%. The review also revealed that all the LGAs had achieved a satisfactory performance for stool adequacy and non-polio AFP rates, which are the two core surveillance indicators (Table 1). Out of the 20 DSNOs, 18 (90%) participated in the study. A review of the expected deliverables for surveillance for WPV by the DSNOs revealed that Integrated Disease Surveillance and Response (IDSR) guidelines and other basic surveillance data tools were available in all the offices of the DSNOs, including a list of focal sites and their prioritization. However, evidence of polio outbreak investigations, supervision reports, and minutes of meetings were available and seen in only 11, 28, and 33% of the DSNO offices, respectively. Additionally, the trend of AFPs identified was documented and seen in only 44% of the offices of the DSNOs. Similarly, a surveillance work plan and trend for IDSR diseases were present and seen in only 39% of the DSNO offices (Table 2).

**Table 1:** Core surveillance indicators for polio eradication in Bauchi state, 2012–2016

	2012	2013	2014	2015	2016
Total population	5715,292	6,909,612	6,110,539	6,318,297	6,53,3157
Population < 15 years of age	2,720,479	2,812,975	2,908,616	3,007,509	3,109,783
Timeliness of reporting (%)	92%	95%	96%	98%	99%
Completeness of reporting (%)	92%	98%	100%	100%	100%
Expected AFP cases	27.2	28.1	29.1	30.1	32.2
No. of AFP reported	240	246	291	534	332
AFP detection rate	9.7	14	15.1	20.7	24.5
NPAFP rate	9.7	14	15.1	20.7	24.5
Stool adequacy (%)	97%	100%	100%	100%	100%
LGAs meeting both criteria (%)	92%	100%	100%	100%	100%
Confirmed WPV cases	6	0	0	0	0

*AFP* acute flaccid paralysis, *LGA* Local Government Area, *NPAFP* non-polio acute flaccid paralysis, *WPV* wild poliovirus

**Table 2:** Distributions of expected deliverables at the 18 Disease Surveillance and Notification Officer (DSNO) offices in Bauchi state, 2016

Variables	Present, *n* (%)
Visitors’ books seen with action point	17 (94.4)
Supervisory book seen	14 (77.8)
IDSR guidelines seen	18 (100.0)
Map of ward seen	18 (100.0)
Map of LGA seen	16 (88.9)
Poster seen	18 (100.0)
Trend of diseases in the LGA	10 (55.6)
Profile of the LGA	9 (50.0)
Trend of AFP in the LGA	8 (44.4)
Trend of IDSR diseases	7 (38.9)
Term of Reference for DSNOs	14 (77.8)
List of focal persons	18 (100.0)
Surveillance work plan available	11 (61.1)
Supervisory plan seen	7 (38.9)
List of health facilities in the LGA	15 (83.3)
List of reporting sites	16 (88.9)
List of informants	15 (83.3)
AFPLG001–4 seen	18 (100.0)
AFP C101	18 (100.0)
AFPF001	15 (83.3)
Polio outbreak investigation	2 (11.1)
Supervisory reports	5 (27.8)
Minutes of meeting	6 (33.3)

*AFP* acute flaccid paralysis, *IDSR* Integrated Disease Surveillance and Response, *LGA* Local Government area

The authentication of reports of the 31 AFP cases investigated revealed discrepancies in 39% for the birthday, 26% for the birth month of the child, 23% for the date of onset of paralysis, and 23% for the date of the investigation. All the mothers interviewed correctly mentioned the name of the child investigated for AFP, the sex of the child, and the name of the community. The mothers correctly mentioned the age of the child (93.5%), date of onset (80.6%), date of investigation (77.4%), stool adequacy (83.9%), and location (87.1%) (Table 3).

**Table 3:** Authentication of 31 acute flaccid paralysis (AFP) case data investigated for wild poliovirus (WPV) as reported by mothers in Bauchi state, 2016

Variables	Present, *n* (%)
Name	31 (100.0)
Birthday	19 (61.3)
Birth month	23 (74.2)
Sex	31 (100.0)
Age	29 (93.5)
Community	31 (100.0)
Date of onset	25 (80.6)
Date of investigation	24 (77.4)
Stool adequacy	26 (83.9)
Place	27 (87.1)

Only 85 (67%) of the designated focal AFP sites were reached and clinicians interviewed. All 85 (100%) of the clinicians (AFP focal persons) at the focal sites had received training on AFP surveillance in the last 2 years (2014 and 2015) before the study. Only 7 (20%) of the 35 clinicians at the non-focal AFP sites had received training on AFP surveillance in the last 2 years. Of the 85 clinicians at the focal sites and 35 at the non-focal sites who were interviewed, 60 (71%) at the focal sites and 17 (39%) at non-focal sites had adequate knowledge (good and very good knowledge) of case definitions of AFP, and 75 (88%) and 28 (65%) for stool collection for investigation among staff, respectively (Table 4).

**Table 4:** Knowledge of clinicians (acute flaccid paralysis (AFP) focal persons) on case definition of AFP and procedures for stool sample collection

Knowledge grade	Case definition of AFP, *n* (%)		Stool sample collection, *n* (%)	
	Focal sites	Non-focal sites	Focal sites	Non-focal sites
Poor knowledge	15 (17.6%)	14 (32.6%)	7 (8.2%)	6 (14.0%)
Fair knowledge	10 (11.8%)	12 (27.9%)	3 (3.5%)	9 (20.9%)
Good knowledge	30 (35.3%)	9 (20.9%)	28 (32.9%)	10 (23.30%)
Very good	30 (35.3%)	8 (18.6%)	47 (55.3%)	18 (41.9%)
Total	85 (100.0%)	43 (100.0%)	85 (100.0%)	43 (100.0%)

Records of trends of disease reported on IDSR, terms of reference for informants, contacts of the informants, and completed forms AFP 001 to AFP 003 were below optimum at the focal sites (Table 5). The majority of the non-focal sites did not have guidelines for IDSR and the AFP 001 and 003 forms (Table 5). Of the 38 mothers or caregivers interviewed, 16 (42%) did not remember the day or month the investigation for the AFP was conducted. Ninety-five percent of the caregivers (mothers) gave the correct number of stool samples, and 40% mentioned that the samples were collected 24 h apart. Feedback was not given to 26 (68%) of the caregivers. The majority (79%) of the community leaders interviewed were aware of AFP and knew that the stool was the specimen needed for investigation of the case, but 21% did not know to whom they needed to report a case of AFP in their community.

**Table 5:** Expected deliverables for acute flaccid paralysis (AFP) surveillance at the focal sites and non-focal sites in Bauchi state, 2016

Variables	Focal site *n* = 85	Non-focal site *n* = 43
Register available	85 (100.0%)	42 (97.7%)
Register seen	85 (100.0%)	42 (97.7%)
Guideline available	77 (90.5%)	16 (37.2%)
Visitors book seen	85 (100.0%)	39 (90.7%)
Action point seen in visitors	84 (98.8%)	35 (81.4%)
Map seen	79 (92.9%)	35 (81.4%)
Poster seen	84 (98.8%)	40 (93.0%)
Trend of diseases	36 (42.4%)	11 (25.6%)
Terms of reference	64 (75.3%)	N/A
Surveillance calendar seen	82 (96.5%)	31 (72.1%)
List of informants	80 (94.1%)	N/A
Contacts of informants	63 (74.1%)	N/A
AFP 001	63 (74.1%)	11 (25.6%)
AFP 002	61 (71.2%)	N/A
AFP 003	51 (60.0%)	5 (11.6%)
IDSR forms	47 (55.7%)	7 (16.3%)
Training folder	69 (81.2%)	N/A

*IDSR* Integrated Disease Surveillance and Response, *N/A* not applicable

## Discussion

The results of this study on the evaluation of quality surveillance revealed that there was a functional and sensitive surveillance system for polio eradication in the state, evident by the high AFP detection and non-polio AFP rates between January 2012 and June 2016 at both state and LGA levels. A highly sensitive surveillance system is required for polio because it is a disease targeted for eradication, and the desire is not to miss any case of AFP that could have been caused by WPV. The importance of the highly sensitive system is to ensure prompt investigation for the disease as noted by WHO [16].

The results show that the polio surveillance system had achieved its key objectives in both the state and LGAs since each had met and maintained the two core surveillance indicators for polio eradication since 2012. Although the state identified the last confirmed case of WPV1 in September 2013 and WPV3 in November 2011, it had remained free of any polio-compatible disease for 4 years which can be credited to the good and functional surveillance system in the state. The achievement is also an indication of an efficient system supporting the interruption of WPV and, as such, the state might be confident of the true absence of WPV [17].

Despite the good results shown for the state, at the operational level, and particularly at the non-focal sites in the study, it was revealed that some of the critical elements for the quality of the surveillance system for polio eradication and eventual certification of polio-free states were deficient. These key elements are knowledge and documentation, and are not mutually exclusive; documentation depends on knowledge of AFP and the skills of the reporting procedures of the officer. They are key determining factors for the completeness, correctness, and reliability of the data. Good knowledge of case definition of the disease enables early detection and prompt investigation. The importance of documentation on the other hand cannot be overemphasized; it is the documentation that provides evidence that efforts have been made to search for WPV and that the virus was absent. Pomerai et al. in their study on evaluation of AFP surveillance in the Bikita district of Masvingo Province in Zimbabwe noted that failure of detection of AFP was due to a lack of the knowledge of the healthcare workers on its symptoms [18].

Documentation is also affected by the motivation and attitude of the public health official. For example, to elicit prompt action, the report must be sent promptly; thus, failure to send a well-documented report on time will not elicit the expected result, and this is dependent on the motivation and attitude of the officer responsible for the task. Several factors affect staff motivation and attitude towards their assigned duties, including surveillance for polio. Studies in African countries show a functional AFP surveillance system that operates despite challenges such as chronic insecurity and inaccessibility, and a lack of capacity and infrastructure [19–25]. Similar studies in Kenya in 2012 observed and reported deficiencies at multiple levels of the health system and were most commonly related to the challenges of funding, training, and supervision [26]. These results corroborate the findings from our study, where capacities at the non-focal sites were a major challenge.

The authentication of reports of AFP investigated revealed discrepancies in the birthday, the birth month of the child, the date of onset of paralysis, and the date of the investigation, indicating problem with both knowledge and documentation by the healthcare workers. This information was collected in retrospect and could had been subject to recall bias. The poor documentation in our study might be one of the important pointers to the outbreak of WPV in Nigeria in June 2016. The genetic sequencing of the outbreak that occurred in Borno state in August 2016 after 2 years of absence suggested that the new cases were most closely linked to a wild poliovirus strain that was last detected in the state in 2011 [27].

Bauchi state, our study site, has been host to some of the displaced persons from Borno state, also putting the state at risk for outbreaks of WPV. Poor knowledge, documentation, and archiving by the LGA DSNOs means that the state could have missed cases of WPV. Furthermore, one of the core assignments of the certification committee in all regions is to review documentation to verify the absence of wild poliovirus [28]. It serves as the critical basis for quality of the entire system. The documentation acts as the sum of the evidence for the knowledge of the operation of the entire surveillance system. Good and complete documentation is a proxy indicator of the quality of the system. Poor documentation, on the other hand, translates into the possibility of missing vital information leading to wild poliovirus being overlooked, either in the past or the future. Documentation is also a proxy indicator of the knowledge of the responsible officers in the polio eradication initiative. The implication is that people with poor knowledge of the requirements may not document the activities correctly. In our study, it was evident that there are gaps in the knowledge of the key operational staff at the health facility and at community levels on the requirements for polio eradication. For example, poor knowledge of case definition for AFP, which is the mainstay for wild poliovirus surveillance, and poor knowledge of when and how to collect stool sample will have a grave consequence for the surveillance system. The lack of knowledge of case definition of AFP seen in our study in the non-focal sites might contribute to overlooked wild poliovirus in the state. The practice of assigning some health facilities as focal or non-focal sites has its advantages and disadvantages. Whereas the designation is based on criteria such as patient volume and special services, it is possible for patients with AFP from wild poliovirus infection to visit the non-focal sites where knowledge about the disease is poor, mainly due to a lack of training, and such patients will be missed.

Mothers of children detected with AFP were active participants in the polio eradication process, particularly in stool sample collection which is the mainstay for investigating a case with suspected WPV. The knowledge of the mothers on how and when to collect the stool sample is very important and will have an impact on the quality of the AFP surveillance. Our study indicated poor knowledge on stool collection among the caregivers. The paucity of knowledge of the mothers on the correct procedure for stool sample collection casts doubts on the quality of the stool samples collected by them. The stool samples not collected according to the standard procedure will not provide the information needed for timely detection of an outbreak of AFP caused by WPV, leading to the erroneous conclusion of absence of WPV [29, 30]. Similarly, the participation of the community in polio surveillance is very important; people can only participate effectively if they have the requisite knowledge, especially where and to whom to report a case of AFP. The community leaders in our study had poor knowledge regarding to whom they should report a case of AFP. The knowledge gaps show that the clinicians, who are the key actors of the polio eradication program, are not doing enough to educate the public on the disease and the eradication program.

Our study had the following limitations. Firstly, we were not able to interview all the clinicians in the selected facilities because some of the facilities in rural areas had only a few trained staff and they were absent at the time of the visit of the research team. This could had been circumvented with prior notice to the prospective respondents. Secondly, authentication of the documented information on a child identified with AFP from the caregivers required their ability to remember the event that took place in the past 3 years. The data could have been affected by recall bias. Thirdly, because of the time span selected, attrition of study staff, without good handover, affected the availability of some of the vital documents for assessment.

Our study shows some gaps in documented evidence at the LGA level, at focal and non-focal sites in the state, feedback to caregivers of children investigated for AFP, and knowledge of the staff at operational levels for AFP surveillance needed for certification of a polio-free Nigeria. The WHO consultants in the polio eradication unit should update the knowledge of the health facility staff on AFP surveillance, especially at the operational level. Additionally, the WHO consultants should provide all the essential materials and tools for documentation of AFP surveillance and to ensure their judicious use at the operational level. The primary healthcare development agency of Bauchi state should conduct aggressive public awareness campaigns on the signs and symptoms of AFP, including surveillance for it.

## Conclusion

Our study revealed a gap in the quality indicators for polio eradication in the state, especially knowledge and documentation for AFP surveillance at the operational level. The state surveillance unit should update the knowledge of the DSNOs and the focal persons, conduct regular sensitization of clinicians and community informants, and timely and adequate supply of reporting tools; ensuring their judicious use will improve AFP surveillance in the state.

## Abbreviations

AFP: Acute flaccid paralysis
DSNO: Disease Surveillance and Notification Officer
IDSR: Integrated Disease Surveillance and Response
LGA: Local Government Area
WHA: World Health Assembly
WHO: World Health Organization
WPV: Wild poliovirus
